# Holistic Evaluation of the Gut Microbiota through Data Envelopment Analysis: A Cross-Sectional Study

**DOI:** 10.1016/j.cdnut.2024.104469

**Published:** 2024-09-27

**Authors:** Taizo Matsuki, Sho Nakamura, Minami Nishiyama, Hiroto Narimatsu

**Affiliations:** 1 Graduate School of Health Innovation, Kanagawa University of Human Services, Tonomachi, Kawasaki-ku, Kawasaki, Kanagawa, Japan; 2 Cancer Prevention and Control Division, Kanagawa Cancer Center Research Institute, Asahi-ku, Yokohama, Kanagawa, Japan; 3 Department of Medical Genetics, Kanagawa Cancer Center, Nakao, Asahi-ku, Yokohama, Kanagawa, Japan

**Keywords:** microbiota, cross-sectional studies, health behavior, gastrointestinal tract, personalized intervention, data envelopment analysis

## Abstract

**Background:**

The gut microbiome plays a crucial role in human health, but maintaining a healthy gut microbiome remains challenging. Current approaches often focus on individual components rather than providing a holistic assessment.

**Objectives:**

To introduce and evaluate a novel approach using data envelopment analysis (DEA) for assessing gut microbiota efficiency and identifying potential targets for personalized interventions.

**Methods:**

We conducted a cross-sectional analysis of 577 participants from the Kanagawa “ME-BYO” Prospective Cohort Study. Lifestyle factors and gut microbiota composition were assessed. DEA was employed to calculate an efficiency score for each participant, incorporating multiple inputs (lifestyle factors) and outputs (gut microbiotas). This score represents how efficiently an individual’s lifestyle factors contribute to their gut microbiota composition. Tobit regression analysis was used to assess associations between efficiency scores and demographic and health-related factors.

**Results:**

The mean efficiency score was 0.86, with 14.2% of participants classified as efficient. Efficiency scores showed positive correlations with alcohol intake and Faith's phylogenetic diversity. Tobit regression analysis revealed significant associations between efficiency scores and sex, fat intake, and yogurt consumption. DEA identified specific targets for improving gut microbiota composition in inefficient individuals.

**Conclusions:**

This study demonstrates the potential of DEA as a tool for evaluating gut microbiota efficiency and providing personalized recommendations for microbiota optimization. This approach could lead to more effective strategies for optimizing gut health across diverse populations.

## Introduction

The human gut microbiome consists of ∼300–500 species of bacteria, amounting to 100 trillion bacteria [1]. These bacteria interact in complex ways that affect our health [2,3]. The gut microbiome composition varies among individuals and is influenced by factors such as lifestyle, diet, birth method, maternal microbiome, sex, and geographical location [2,4]. Notably, the gut microbiome can impact our health, whereas host factors concurrently shape its composition. Associations between the gut microbiome and various conditions, including metabolic diseases such as diabetes mellitus; immune-related disorders such as inflammatory bowel disease; and cardiovascular diseases, cancer, and central nervous system disorders, have been observed [3,5,[6], [7], [8], [9]]. The gut–brain axis, for instance, links the microbiome to brain function and mental health [2,6,7]. Consequently, maintaining a healthy gut microbiome has become an important measure, alongside conventional practices such as a healthy lifestyle, good hygiene, adequate sleep, and a balanced diet [6,9,10].

A favorable gut microbiome composition is characterized by an abundance and high species diversity of beneficial bacteria [11]. Beneficial microorganisms, including lactic acid bacteria (*Lactobacillus* genus), *Bifidobacterium* (*Bifidobacterium* genus), and *Saccharomyces boulardii* (a tropical yeast), offer protective effects [12]. They produce short-chain fatty acids, such as acetic acid, butyric acid, and lactic acid, which enhance host immunity and protect against pathogenic bacteria by supporting intestinal epithelial cells. Lifestyle choices can influence the maintenance and growth of these beneficial bacteria [7]. For example, stress can decrease the abundance of *Lactobacillus*, whereas smoking can increase the presence of potentially harmful bacteria such as *Bacteroides-Prevotella*, which may contribute to inflammatory diseases such as Crohn's disease [13,14]. Consuming probiotics and prebiotics through diet can promote the abundances of beneficial bacteria and increase microbial diversity [15,16]. Probiotics are live microbial supplements derived from fermented foods, whereas prebiotics are non-digestible carbohydrates such as oligosaccharides, dextrin, and inulin, which act as fermentable dietary fibers, thereby promoting the growth of beneficial bacteria [7,[16], [17], [18]].

Although lifestyle factors significantly influence gut microbiome composition, they do not fully explain the observed diversity [19]. We use the term “constitution” to account for this unexplained variation. We are particularly interested to assess how lifestyle affects the acquisition of a preferable gut microbiome composition, which we refer to as gut microbiome efficiency. Some individuals may possess a favorable gut microbiome despite having an unhealthy lifestyle, whereas others may have an unfavorable microbiome despite careful lifestyle choices. We hypothesized that the former group has better gut microbiome efficiency compared with that of the latter. To evaluate this efficiency, we propose using data envelopment analysis (DEA) [20].

DEA is commonly applied in operations research, particularly in management engineering and economics, for measuring and comparing the productivity efficiency of decision-making units (DMUs), such as management entities (for example, retail stores or facilities) [20]. A DMU that produces greater output relative to input (higher output/input value) is considered more efficient. A key advantage of this method is its ability to calculate the distance and direction from the efficiency frontier for inefficient DMUs, enabling the consideration of compensatory measures to improve efficiency [21,22].

DEA can also be used for evaluating disease risk by considering each individual as a DMU. For example, the efficiency score of a normotensive population, calculated using salt intake and physical activity as input and blood pressure as output, has been found to be associated with future onset of hypertension [23]. Similarly, by treating each individual as a DMU, with their lifestyle as input and their gut microbiota composition as output, the calculated efficiency score can indicate gut microbiota efficiency. Evaluation of the gut microbiota efficiency using DEA can provide personalized suggestions to every inefficient DMU. The microbiota that is most effective in improving gut microbiota composition can be identified, thereby allowing individuals to select suitable prebiotic or probiotic products in addition to conventional measures such as a healthy lifestyle, which can be challenging to maintain in the long term. Another advantage of this technique is its ability to handle multiple input and output variables that are generally not comparable with each other. This enables us to provide personalized and multifaceted suggestions for improving health and the gut microbiota environment based on the reference peers, which consist of DMUs belonging to the efficiency frontier. This approach is contradictory to conventional analyses that assess average behavior among the population of interest.

On the basis of these considerations, in this study, we aimed to explore the potential of using DEA for evaluating the gut microbiota environment and identifying the specific microbiotas required for a relatively healthy gut environment in each individual. Identifying the required microbiota through assessing gut microbiota efficiency enables us to provide personalized interventions, such as supplementary pre- or probiotics, to improve the microbiota composition. The results of this study could provide a scientific basis for developing tailored approaches to optimize gut health based on an individual's unique microbiota profile and environmental factors.

## Methods

### Study population

We conducted a cross-sectional analysis using the baseline data obtained from the Kanagawa “ME-BYO” Prospective Cohort Study (ME-BYO cohort) that is a part of the Japan Multi-Institutional Collaborative Cohort Study. Details of these studies are described elsewhere [24,25]. The ME-BYO cohort is a genomic cohort study conducted at the Kanagawa Cancer Center Research Institute in Kanagawa Prefecture, Japan. The baseline survey began in 2016 and is still ongoing. Participants were local residents aged 18–95 y and living or working in Kanagawa. This study included participants recruited by the end of March 2022 (v.2206). All participants provided written informed consent, and the Institutional Review Board of Kanagawa Cancer Center approved all study procedures (28KEN-36).

### Measurements

Height and weight were measured to calculate BMI. Lifestyle information and health conditions were collected using a self-administered questionnaire. Daily nutritional intake was evaluated using a validated food frequency questionnaire (FFQ) and adjusted for total energy intake using the residual method [[26], [27], [28]]. We selected nutrients, such as omega-3 and omega-6 unsaturated fatty acids (n3PUFA, n6PUFA), MUFAs, SFAs, and soluble and insoluble dietary fiber (SDF, IDF), which are beneficial to the human gut microbiota [2,7,15]. Salt intake was calculated by multiplying sodium intake by 2.54. We included the daily consumption frequency of yogurt, natto (fermented soybean), seaweed, fried foods, and coffee from the FFQ. Daily alcohol consumption was assessed in units, with 1 unit corresponding to 21 g alcohol. Smoking status was based on the current number of cigarettes smoked per day. Self-reported average daily sleep time was obtained, with 10 missing values imputed with the mean and median of 6.5 h. Physical activity was estimated as metabolic equivalent hours per day (MET-h/d), with physical activity ≥3 METs assessed as moderate-to-vigorous physical activity (MVPA) [29]. Details of calculating MVPA are provided in the Supplemental Methods. Antibiotic and probiotic intakes within 2 and 1 wk before fecal sample collection, respectively, were coded as 1, and 0 otherwise. Blood examination results from the baseline survey were also used.

### Acquisition of the microbiota data

Participants collected fecal samples at home using a brush-type kit containing guanidine thiocyanate solution (Techno Suruga Laboratory). The samples were then mailed to the laboratory at room temperature. Detailed methods for obtaining the microbiota data are described in detail elsewhere [30,31]. DNA was extracted using a DNeasy PowerSoil Kit (Qiagen), following the manufacturer’s instructions. Short-amplicon 16S rRNA gene sequencing targeting the V1–V2 region was performed using 27F forward and 338R reverse primers. The sequences were clustered using QIIME2 pipeline v.2020.8 [32]. After clustering, taxonomic assignment was performed using the robust taxonomy simplifier for SILVA (arts-SILVA), which was developed from the 16S rRNA taxonomy dataset based on SILVA 138 [33]. From 450 available operational taxonomic units (OTUs) (450 genera), we selected 5 genera (*Bacillus*, *Bifidobacterium*, *Lactobacillus*, *Lactococcus*, and *Streptococcus*) known to be beneficial for humans based on recent literature and their widespread availability in the Japanese market as of March 2022 [34]. These OTUs were chosen to facilitate future intervention studies based on the results of this study, thereby ensuring that the general population can easily obtain them as food products or supplements. In addition, Faith's phylogenetic diversity (FPD) was calculated from 10,000 sequencing depth with 10 iterations in QIIME2, and the mean value was used as a measure of alpha-diversity [35].

### DEA

We used DEA to include multiple inputs and outputs without requiring specification for an a priori function [20]. Each individual was treated as a DMU responsible for converting inputs into outputs. We used the output-oriented variance returns-to-scale Banker–Charnes–Cooper model, where individuals are efficient when output is maximized, whereas inputs are held constant [21,36]. Input variables included 5 nutrients (MUFA, n3PUFA, n6PUFA, SDF, and IDF), alcohol intake, smoking status, MVPA, and sleep time, which are known to affect the human microbiota environment [2,7,37]. Variables that are not beneficial to the microbiotas (alcohol intake and smoking) were inversed to fit the model definition. Alcohol intake and smoking status were subtracted from 5.5 and 90, respectively, for every observation. Outputs were the relative abundances of the selected microbiotas. Variables were preprocessed to fit the non-zero and non-negative constraints in DEA. Details of the transformation used in each variable are described in Supplemental Methods. We checked the correlation between input and output variables using Pearson’s correlation and excluded variables with weak evidence of correlation (*P* > 0.05; Table 1): MUFA, n6PUFA, and MVPA were excluded. We also excluded *Bifidobacterium*, which showed a negative correlation with the input variables, where the correlation should be positive for beneficial bacteria. The efficiency score was calculated using dea function in *Benchmarking* package [38]. We converted the obtained value (inefficiency score) to the efficiency score by taking the reciprocal value, theoretically ranging between 0 and 1. In addition, we calculated efficiency scores in different models by modifying several input and/or output variables in our main model to evaluate the robustness of the results. Details of this analysis are provided in Supplemental Methods.

**TABLE 1:** Correlation between candidate input and output variables for DEA.

Input variables	*Bacillus*		*Bifidobacterium*		*Lactobacillus*		*Lactococcus*		*Streptococcus*		FPD	
	*r* (95% CI)	*P* value	*r* (95% CI)	*P* value	*r* (95% CI)	*P* value	*r* (95% CI)	*P* value	*r* (95% CI)	*P* value	*r* (95% CI)	*P* value
Alcohol inake^1^ (21 g/d)	0.08 (0.00, 0.16)	0.044	0.07 (–0.01, 0.15)	0.086	0.09 (0.00, 0.17)	0.039	0.02 (–0.06, 0.10)	0.664	0.22 (0.14, 0.29)	<0.001	0.02 (–0.06, 0.10)	0.655
MUFA	0.004 (–0.08, 0.09)	0.919	–0.05 (–0.13, 0.03)	0.258	–0.01 (–0.09, 0.07)	0.839	0.03 (–0.05, 0.11)	0.514	–0.02 (–0.10, 0.06)	0.633	0.01 (–0.07, 0.09)	0.750
n3PUFA (mg/d)	–0.02 (–0.10, 0.06)	0.581	–0.11 (–0.19, –0.03)	0.006	0.02 (–0.06, 0.10)	0.625	0.05 (–0.03, 0.13)	0.260	–0.01 (–0.09, 0.08)	0.876	0.09 (0.01, 0.17)	0.029
n6PUFA (mg/d)	0.01 (–0.07, 0.09)	0.868	–0.04 (–0.12, 0.04)	0.350	0.004 (–0.08, 0.09)	0.915	–0.03 (–0.11, 0.05)	0.510	–0.03 (–0.11, 0.05)	0.510	0.07 (–0.01, 0.15)	0.084
Soluble dietary fiber (g/d)	0.15 (0.07, 0.23)	<0.001	–0.07 (–0.15, 0.01)	0.090	0.07 (–0.01, 0.15)	0.082	0.09 (0.00, 0.17)	0.038	0.13 (0.05, 0.21)	0.002	0.18 (0.10, 0.26)	0.000
Insoluble dietary fiber (g/d)	–0.02 (–0.10, 0.07)	0.691	0.02 (–0.06, 0.10)	0.604	–0.09 (–0.17, –0.00)	0.040	0.01 (–0.07, 0.09)	0.765	0.01 (–0.07, 0.09)	0.827	–0.01 (–0.09, 0.08)	0.897
Smoking^1^ (*n*/d)	0.04 (–0.05, 0.12)	0.381	–0.04 (–0.12, 0.04)	0.329	–0.03 (–0.12, 0.05)	0.414	–0.01 (–0.09, 0.07)	0.811	0.02 (–0.06, 0.10)	0.656	0.04 (–0.04, 0.12)	0.361
MVPA (METs-h/d)	0.02 (–0.06, 0.11)	0.565	0.02 (–0.06, 0.10)	0.647	0.08 (0.00, 0.16)	0.049	0.01 (–0.07, 0.09)	0.769	0.01 (–0.07, 0.10)	0.738	0.06 (–0.02, 0.14)	0.168
Total sleep time (h/d)	0.08 (0.00, 0.16)	0.044	0.07 (–0.01, 0.15)	0.086	0.09 (0.00, 0.17)	0.039	0.02 (–0.06, 0.10)	0.664	0.22 (0.14, 0.29)	<0.001	0.02 (–0.06, 0.10)	0.655

Abbreviations: CI, confidence interval; DEA, data envelopment analysis; FPD, Faith’s phylogenetic diversity; MET, metabolic equivalent; MUFA, mono-unsaturated fatty acids; MVPA, moderate-to-vigorous physical activity; n3, omega-3; n6, omega-6; PUFA, poly-unsaturated fatty acids.

Data indicate Pearson's correlation coefficient.

1 Inversed variables.

### Statistical analysis

All statistical analyses were performed using R v.4.1.2 (R Core Team) [39]. Correlations between the calculated efficiency score and input/output variables were calculated using Pearson’s product-moment correlation. Given the bound nature of DEA efficiency score (0–1), we performed Tobit regression using vglm function of VGAM package [40]. The lower and upper bounds were set to 0 and 1, respectively. Explanatory variables included age, sex, BMI, estimated glomerular filtration rate (eGFR), hemoglobin, glycated hemoglobin A_1c_ (HbA1c), nutritional intakes, FFQ items, and antibiotic/probiotic intake. Nutritional intake included total energy intake, fat intake, and salt intake, and FFQ items included frequency of coffee, yogurt, natto, seaweeds, and fried food intake. Missing values in BMI (*n* = 8), eGFR (*n* = 40), HbA1c (*n* = 27), and hemoglobin (*n* = 29) were imputed using the multivariate imputation by chained equations with a predictive mean matching method using mice function in *mice* package [41]. We used 20 imputations with 50 iterations each. All variables utilized in Tobit regression were used for imputation. In addition, daily carbohydrate, protein, and triglyceride intake, total cholesterol, high-density lipoprotein cholesterol, total protein, albumin, white blood cell count, and platelet count were used as auxiliary variables. To avoid overfitting and improve stability, we used quickpred function in *mice* package for selecting appropriate predictors for each variable with missing data. Quickpred function was set to include predictors with a minimum correlation of 0.1 with the variable to be imputed and a minimum proportion of usable cases of 0.25. Additionally, key variables of interest (age, sex, efficiency score, and output variables in DEA) were forcibly included in all imputation models, regardless of their correlation. The plausibility of imputed values was assessed by comparing distributions of observed and imputed data (Supplemental Figure 1). Pooled results from multiple imputed datasets were obtained according to Rubin's rules using MIcombine function in *mitools* package [42,43]. A complete case analysis was conducted as a sensitivity analysis for evaluating the robustness of our findings to the missing at random assumption underlying the multiple imputation approach. Furthermore, these explanatory variables were used to perform multivariate Tobit regression. Variable selection using a backward elimination procedure was conducted using a stepwise algorithm based on *P* value with a cut-off of 0.05. The variables selected in ≥50% datasets were retained in the final model. The variables (selected times) in the final model were sex (20), BMI (20), hemoglobin (20), total energy intake (20), fat intake (20), salt intake (20), and frequency of yogurt intake (20). Multicollinearity was assessed by calculating the variance inflation factor (VIF) for each imputed dataset by ordinary least square and averaging the results. The largest mean VIF was 1.46 (sex), indicating no potential multicollinearity issues.

## Results

We calculated the efficiency scores of 577 participants with available gut microbiota data out of 3409 ME-BYO cohort participants, using DEA. Participant characteristics are shown in Table 2. The mean (SD) age was 58.6 (14.6), ranging between 21 and 84 years, and 366 of the 577 (63.4%) participants were female.

**TABLE 2:** Characteristics of the study participants.

Characteristics	*n* = 577
Age (y)	58.6 (14.6)
Sex (female, *n* [%])	366 (63.4)
Daily nutritional intake	
Energy intake (kcal)	1545 (437)
Fat intake (g)	47.8 (12.0)
Saturated fatty acids (g)	12.1 (3.1)
MUFA (g)	17.2 (4.4)
n3PUFA (g)	2.2 (0.6)
n6PUFA (g)	10.9 (3.0)
Total dietary fiber (g)	11.1 (3.3)
Soluble	2.1 (0.7)
Insoluble	8.2 (2.3)
Salt intake (g)	4.4 (1.3)
Alcohol intake^1^ (unit)	0.3 (0.7)
Smoking^2^ [*n* (%)]	188 (32.6)
Daily number of cigarettes smoked	5.3 (10.1)
MVPA (METs-h/d)	12.0 (10.6)
Sleep time (h/d)	6.5 (1.0)
Microbiota^3^	
*Bacillus* [‰, median (IQR)]	1.81 (0.78, 3.09)
*Lactobacillus* [‰, median (IQR)]	1.33 (0.37, 6.74)
*Lactococcus* [‰, median (IQR)]	0.31 (0.16, 0.88)
*Streptococcus* [‰, median (IQR)]	6.46 (2.28, 20.33)
Faith's phylogenetic diversity	30.8 (7.9)

Abbreviations: IQR, interquartile range; MET, metabolic equivalent; MUFA, mono-unsaturated fatty acids; MVPA, moderate-to-vigorous physical activity; n3, omega-3; n6, omega-6; PUFA, poly-unsaturated fatty acids.

Data are shown as mean (SD) unless otherwise specified.

1 One unit corresponds to 21 g alcohol.

2 Currently smoking or have smoked in the past.

3 Values are for 208/577 (36.0%), 236/577 (40.9%), 86/577 (14.9%), 569 (98.6%), and 166/577 (28.8%) participants in *Bacillus*, *Lactobacillus*, *Lactococcus*, and *Streptococcus*, respectively, whose relative abundance was not 0.

The mean (SD) efficiency score was 0.86 (0.11) ranging between 0.54 and 1.00. Correlation analysis between the efficiency score and DEA variables showed that alcohol intake had the highest correlation among input variables [*r*(575) = –0.247; 95% confidence interval (CI): –0.322, –0.169; *P* < 0.001, Table 3]. Among output variables, FPD had the highest correlation [*r*(575) = 0.470; 95% CI: 0.404, 0.532; *P* < 0.001]. A total of 82 of 577 (14.2%) participants were efficient, forming the efficiency frontier. Results from the sensitivity analysis for assessing the robustness of our DEA model, which involved calculating efficiency scores with different combinations of input and output variables (Supplemental Table 1), are shown in Supplemental Figure 2. The correlation coefficients between the efficiency scores of our main and alternative models ranged between 0.84 and 0.99.

**TABLE 3:** Correlation coefficients between efficiency scores and variables used in data envelopment analysis.

Variables	Correlation coefficients (95% CI)	*P* value
Inputs		
n3PUFA	–0.149 (–0.227, –0.068)	<0.001
Soluble dietary fiber	–0.159 (–0.237, –0.078)	<0.001
Insoluble dietary fiber	–0.154 (–0.233, –0.074)	<0.001
Alcohol intake^1^^,^^2^ (unit/d)	–0.247 (–0.322, –0.169)	<0.001
Smoking^2^	–0.093 (–0.174, –0.012)	0.025
Sleep time (h/d)	–0.091 (–0.172, –0.01)	0.028
Outputs		
*Bacillus*	0.192 (0.112, 0.27)	<0.001
*Lactobacillus*	0.331 (0.256, 0.402)	<0.001
*Lactococcus*	0.155 (0.074, 0.233)	<0.001
*Streptococcus*	0.314 (0.239, 0.386)	<0.001
Faith's phylogenetic diversity	0.470 (0.404, 0.532)	<0.001

Abbreviations: CI, confidence interval; n3, omega-3; PUFA, poly-unsaturated fatty acids.

Pearson’s product-moment correlation between efficiency score obtained by output-oriented variance returns-to-scale data envelopment analysis and each variables. The 95% confidence interval is shown in the parenthesis.

1 One unit corresponds to 21 g alcohol.

2 Inversed variables.

The characteristics of participants according to the efficient (efficiency score = 1.0) and inefficient subgroups are shown in Table 4. The proportion of female participants was 36/82 (43.9%) in efficient participants, whereas 330/495 (66.7%) in the inefficient participants. Participants having a comorbid condition seemed to be relatively high in efficient participants; efficient compared with inefficient participants were 12/82 (14.6%) compared with 37/495 (7.5%), 34/82 (41.5%) compared with 94/495 (19.0%), and 9/82 (11.0%) compared with 19/495 (3.8%) for cancer, hypertension, and diabetes, respectively. A total of 3/577 (0.5%), and 15/577 (2.6%) participants were taking antibiotics and probiotics, respectively (Table 4).

**TABLE 4:** Characteristics according to the efficiency score subgroup.

	Entire population (*n* = 577)	Efficient (*n* = 82)	Inefficient (*n* = 495)
Age (y)	58.6 (14.6)	60.9 (15.3)	58.2 (14.5)
Sex [female, *n* (%)]	366 (63.4)	36 (43.9)	330 (66.7)
BMI^1^ (kg/m^2^)	22.6 (3.1)	23.5 (3.5)	22.4 (3.0)
eGFR^1^ (mL/min/1.73 m^2^)	75.4 (16.0)	70.6 (18.0)	76.2 (15.4)
Hemoglobin^1^ (mg/dL)	13.9 (1.4)	13.8 (1.6)	13.9 (1.4)
Blood urea nitrogen (mg/dL)	20.7 (30.4)	16.5 (5.4)	21.4 (32.8)
HbA1c^1^ (%)	5.60 (0.53)	5.66 (0.67)	5.59 (0.51)
Total energy intake (kcal/d)	1545 (437)	1758.8 (768.1)	1509.3 (342.1)
Fat intake (g/d)	47.8 (12.0)	41.6 (12.7)	48.8 (11.6)
Salt intake (g/d)	4.4 (1.3)	3.8 (1.7)	4.5 (1.2)
Food frequency items			
Yogurt	0.2 (0.1, 1.0)	0.5 (0.1, 1.0)	0.2 (0.1, 1.0)
Natto	0.2 (0.1, 0.5)	0.2 (0.1, 0.7)	0.2 (0.1, 0.5)
Seaweed	0.2 (0.1, 0.5)	0.2 (0.1, 0.5)	0.2 (0.1, 0.5)
Fried food	0.2 (0.1, 0.2)	0.2 (0.1, 0.2)	0.2 (0.1, 0.2)
Coffee	1.0 (0.5, 2.0)	1.0 (0.2, 1.0)	1.0 (0.5, 2.0)
Comorbid condition [*n* (%)]			
Cancer	49 (8.5)	12 (14.6)	37 (7.5)
Hypertension	128 (22.2)	34 (41.5)	94 (19.0)
Diabetes	28 (4.9)	9 (11.0)	19 (3.8)
Dyslipidemia	96 (16.6)	18 (22.0)	78 (15.8)
Gastric ulcer	45 (7.8)	11 (13.4)	34 (6.9)
Duodenal ulcer	37 (6.4)	10 (12.2)	27 (5.5)
Colorectal polyps	77 (13.3)	16 (19.5)	61 (12.3)
Medication [*n* (%)]			
Antibiotics	3 (0.5)	1 (1.2)	2 (0.4)
Probiotics	15 (2.6)	3 (3.7)	12 (2.4)

Abbreviations: BMI, body mass index; eGFR, estimated glomerular filtering rate; HbA1c, glycated hemoglobin A_1c_.

Data are shown as mean (SD) unless otherwise specified. Efficient participants have an efficiency score of 1.0, and inefficient participants are the rest of participants.

1 Missing values (BMI, 8; eGFR, 40; hemoglobin, 29; and HbA1c, 27) were imputed using multivariate imputation by chained equations with predictive mean matching.

The results of Tobit regression analysis are shown in Table 5. The efficiency score was associated with sex (female, *β* = –33.9 × 10^3^; 95% CI: –59.8 × 10^3^, –8.1×10^3^; *P* = 0.024), fat intake (per 1 g, *β* = –1.5 × 10^3^; 95% CI: –2.4×10^3^, –0.7 × 10^3^; *P* = 0.001), and yogurt intake (per 1 time/d, *β* = 22.1 × 10^3^; 95% CI: 1.18×10^3^, 42.4 × 10^3^ *P* = 0.033). Results of the sensitivity analysis are shown in Supplemental Table 2.

**TABLE 5:** Results of Tobit regression analysis on efficiency score.

Explanatory variables	Univariate analysis			Multivariate analysis		
	Coefficients	SE	*P* value	Coefficients	SE	*P* value
Age	0.25	0.35	0.474	—		
Sex (female)	–40.59	10.43	<0.001	–33.94	13.17	0.010
BMI^1^ (kg/m^2^)	3.65	1.64	0.038	3.71	1.64	0.024
eGFR^2^ (20 mL/min/1.73 m^2^)	–13.54	6.49	0.051	—		
Hb^2^ (mg/dL)	0.29	3.67	0.939	–10.05	4.28	0.019
BUN (mg/dL)	–0.07	0.19	0.725	—		
HbA1c^1^ (%)	3.07	9.54	0.751	—		
Total energy intake (500 kcal/d)	26.98	6.41	<0.001	0.03	0.01	0.018
Fat intake (g/d)	–1.66	0.42	<0.001	–1.53	0.44	0.001
Salt intake (g/d)	–14.00	3.84	<0.001	–9.37	4.00	0.019
Food frequency (times/d)						
Yogurt	11.21	10.21	0.272	22.09	10.34	0.033
Natto	2.21	11.41	0.847	—		
Seaweed	–22.39	14.81	0.131	—		
Fried foods	–17.51	24.02	0.466	—		
Coffee	–6.90	5.15	0.180	—		
Taking antibiotics (yes)	30.27	71.03	0.670	—		
Taking probiotics (yes)	1.59	31.84	0.960	—		

Abbreviations: BUN, blood urea nitrogen; eGFR, estimated glomerular filtration rate; HbA1c, glycated hemoglobin A_1c_.

Coefficients and SEs are multiplied by 10^3^, where 1.0 corresponds to 0.001 of the efficiency score. Variables with hyphens are dropped in the variable selection procedure.

1 Missing values (BMI, 8; eGFR, 40; hemoglobin, 29; and HbA1c, 27) were imputed using multivariate imputation by chained equations with predictive mean matching.

Table 6 shows the example of the most inefficient DMU using the results from DEA, to explore responsible outputs for improving gut microbiota efficiency, that is, identifying the specific microbiotas required to achieve a relatively healthy gut environment. Compared with the efficient DMUs, the most inefficient DMU required increasing relative abundance of *Bacillus* at 0.01‰, *Lactobacillus* at 0.49‰, *Lactococcus* at 0.16‰, *Streptococcus* at 2.80‰, and FPD at 12.2.

**TABLE 6:** Visualization of the lack in output using lambda values from DEA.

	*Bacillus*	*Lactobacillus*	*Lactococcus*	*Streptococcus*	FDP
Peer 1 (λ = 0.239)	0.277	3.702	3.669	0.308	6.878
Weighted value	0.066	0.884	0.876	0.073	1.643
Peer 2 (λ = 0.273)	0.277	0.616	0.067	0.820	6.357
Weighted value	0.076	0.168	0.018	0.224	1.737
Peer 3 (λ = 0.334)	0.277	2.063	0.067	0.308	6.218
Weighted value	0.092	0.688	0.022	0.103	2.074
Peer 4 (λ = 0.088)	1.739	3.269	0.067	0.308	7.529
Weighted value	0.153	0.287	0.006	0.027	0.661
Peer 5 (λ = 0.062)	1.981	2.611	0.067	0.308	6.084
Weighted value	0.124	0.163	0.004	0.019	0.380
Peer 6 (λ = 0.041)	1.038	2.747	0.067	0.308	7.017
Weighted value	0.004	0.011	0.000	0.001	0.029
Sum of weighted values	0.515	2.202	0.927	0.448	6.524
Most inefficient DMU	0.277	0.616	0.067	0.308	3.509
Lack of output^1^	0.238	1.586	0.860	0.140	3.015
Back-transformed value^2^	0.01‰	0.49‰	0.16‰	2.80‰	12.2

Abbreviations: DEA, data envelopment analysis; DMU, decision-making unit; Faith’s phylogenic diversity.

The lack of output values used in DEA for the most inefficient DMU (efficiency score, 0.54) is shown. Weighed values are obtained by multiplying each output value with λ for each peer.

1 Lack of output = (sum of weighted values) − (values for most inefficient DMU).

2 Value reversed to the corresponding value before performing data transformation as DEA pre-process.

## Discussion

This study utilized DEA to calculate gut microbiota efficiency using cross-sectional data on lifestyle and gut microbiota composition collected from a cohort study on healthy participants. The resulting efficiency score demonstrates potential as an indicator for improving individuals’ gut microbiota environments.

The primary strength of using the DEA-calculated efficiency score is its ability to provide specific values that each participant needs to increase for achieving efficiency [21]. This optimization is performed for each participant, allowing for personalized recommendations for all inefficient individuals. For instance, based on our results, the most inefficient DMU could aim to increase *Lactobacillus* 0.49‰ by consuming *Lactobacillus*-containing food or supplements [44]. Notably, the bacterial species/genera used as output variables in this model are commercially available as probiotics and easily consumable [17,45]. This individualized approach for evaluating the gut microbiota efficiency could potentially be a useful tool for personalized interventions, which is distinct from the conventional approach based on evidence from statistical methods that focus on average population behavior [20,22,36].

Another advantage of our approach is the holistic evaluation of gut microbiota environment using the capability of DEA to simultaneously incorporate multiple inputs and outputs [20]. Our analysis incorporated 6 inputs and 5 outputs concurrently, a feat not achievable by conventional studies that typically assess the associations between environmental factors and gut microbiome components on a one-to-one basis [2,46]. Although some studies employ multivariate regression analysis, these results show only the isolated effects of individual variables without considering complex interactions within the gut microbiome environment. DEA enabled us to simultaneously analyze lifestyle factors and gut microbiome components, an approach not previously reported in the literature.

High gut microbiota efficiency, as evaluated in this study, can theoretically be achieved when output variables are relatively high despite relatively low input variables. Participants with optimal lifestyles would be expected to have healthier gut microbiota environments than those with suboptimal lifestyles. This expectation was reflected in the observed association between the efficiency score and BMI, as well as comorbid conditions. These findings suggest that some individuals may maintain considerable gut microbiota efficiency despite suboptimal lifestyle factors. Individual factors, such as constitutional elements, including genetic or environmental factors not included in the DEA model, may contribute to this efficiency [8,9]. Although it may not represent the conventional application of DEA methodology, inefficient participants could potentially improve their efficiency by modifying factors not included in the input variables. For individuals maintaining healthy lifestyles yet classified as inefficient, options for improvement extend beyond direct supplementation with beneficial microbiota components. These options include taking inulin-type fibers or galactooligosaccharides. Additionally, they may achieve a relatively favorable gut microbiota environment by modifying the intermediary factors discussed above.

Our results align with those of the previous studies demonstrating the influence of age and sex on gut microbiota composition [47,48]. The observed effect of sex on gut microbiota efficiency in our study is consistent with these findings. Additionally, the negative association between high-fat intake and gut microbiota efficiency corresponds with the known effects of high-fat diets on dysbiosis of the gut microbiota, which can lead to lifestyle-related diseases such as obesity, diabetes, and cardiovascular disorders (Table 5) [49]. These observations suggest that the efficiency scores calculated using our DEA model reasonably reflect an individual’s capacity to maintain a healthy gut microbiota environment.

However, this study has several limitations. We were unable to include *Bifidobacterium*, a well-known beneficial bacterium [1,6,8], in our DEA model owing to its unexpected negative correlation with n3PUFA. This may be attributed to confounding effects from high-fat foods in the FFQ used for estimating n3PUFA intake, or to the use of relative abundance data [2,50], which can yield different associations compared with those of absolute abundance measurements.

The sensitivity analysis of our DEA model demonstrates its overall robustness when considering different combinations of input and output variables. Although efficiency scores were lower in alternative models than in the main model, the high correlation coefficients between the efficiency scores of our main and alternative models suggest that these variations do not substantially affect the interpretation of our results. However, the selection of input and output variables can influence the DEA results, and our choices were based on biological relevance and data availability.

Additional limitations include potential selection bias because our study population was comprised of volunteers from a restricted geographic area. The cross-sectional nature of our data does not account for daily and seasonal fluctuation in gut microbiota composition [51], and the self-reported nature of some data introduces potential bias. Lastly, although we present the possibility of improving gut microbiota efficiency through lifestyle modifications or prebiotic/probiotic supplementation, longitudinal studies are necessary to confirm the efficacy of such interventions.

In conclusion, the efficiency score calculated by DEA is a promising tool for evaluating gut microbiota efficiency. Future longitudinal studies may enable the development of personalized interventions using this efficiency score by identifying inefficient individuals who require improvement in their gut microbiota composition. This approach could contribute to the identification of effective targeting strategies for optimizing gut health across diverse populations.

## Author contributions

The authors’ responsibilities were as follows – TM, SN: designed research, conducted research, analyzed data, and wrote paper; SN, HN: provided essential materials; SN: had primary responsibility for final content; and all authors: read and approved the final manuscript.

## Funding

This study was supported by Grants-in-Aid for Scientific Research for Priority Areas of Cancer (No. 17015018) and Innovative Areas (No. 221S0001) and by the Japan Society for the Promotion of Science (JSPS) KAKENHI Grant [No. 16H06277, 22H04923 (CoBiA) and 17K17614] from the Japanese Ministry of Education, Culture, Sports, Science and Technology.

The Grants-in-Aid for Scientific Research (KAKENHI) mentioned in our article include:

- Priority Areas of Cancer (No. 17015018)

- Innovative Areas (No. 221S0001)

- JSPS KAKENHI Grants (No. 16H06277, 22H04923 (CoBiA), and 17K17614).

These research grants are part of funding programs administered by the Japan Society for the Promotion of Science (JSPS).

## Data availability

Data cannot be shared publicly due to ethical restrictions. Data described in the manuscript will be made available upon application and approval from the Kanagawa “ME-BYO” Prospective Cohort Study office (contact via the corresponding author) for researchers who meet the criteria for data sharing.

## Conflict of interest

All authors declare that there are no potential conflicts of interest to declare.
