# Supplementary material for: Holistic Evaluation of the Gut Microbiota through Data Envelopment Analysis: A Cross-Sectional Study
Source: Curr Dev Nutr. 2024 Sep 27;8(11):104469. doi: 10.1016/j.cdnut.2024.104469 (PMC11550754; doi:10.1016/j.cdnut.2024.104469)
Supplement: Multimedia component 1 [file mmc1.pdf]

Holistic evaluation of the gut microbiota through data envelopment analysis: A cross-sectional study
Matsuki T, Nakamura S, et al.

Online Supplementary Material

TABLE OF CONTENTS

Supplementary Methods ··········· 2

Supplementary Table 1. Variables used in each sensitivity analysis models ··········· 5

Supplementary Table 2. Results of sensitivity analysis of Tobit regression analysis on efficiency score ··········· 6

Supplementary Figure 1. Distribution of observed vs imputed values ··········· 7

Supplementary Figure 2. Comparison of efficiency scores among five models of the DEA ··········· 8

Supplementary Methods

Calculation of physical activity

We estimated physical activity in metabolic equivalent hours per day (MET-h/day), considering both daily and leisure-time activities with MET values ≥ 3. For daily physical activity, participants reported the average time spent per day in eight categories for each of the following daily activities including heavy physical work (assigned 4.5 METs) and walking (3.0 METs). Average time categories (assigned time in hours) were as follows: none (0), < 1 h/day (0.5), 1–< 3 h/day (2.0), 3–< 5 h/day (4.0), 5–< 7 h/day (6.0), 7–< 9 h/ day (8.0), 9–< 11 h/day (10.0), and ≥ 11 h/day (11.0). Daily physical activity was estimated by multiplying the average time with the assigned MET value for each activity. For leisure-time activity, participants reported the frequency and average duration for each activity: light-intensity physical activity (e.g., walking and golf, assigned 3.4 METs as the median value), which did not induce harder-than-normal breathing, moderate-intensity physical activity that induced somewhat harder-than-normal breathing (e.g., jogging and swimming, 7.0 METs assigned as the median value), and vigorous-physical activity that induced harder-than-normal breathing (e.g., marathon running and martial arts, 10 METs assigned as the median value). The frequency and average duration were reported in five and six categories, respectively. The frequency categories (assigned daily average frequency) were as follows: none (0), 1–3 times/month (2/30), 1–2 times/week (1.5/7), 3–4 times/week (3.5/7), and ≥ 6 times/week (6/7). The average duration categories (assigned time in hours) were as follows: < 30 min (15/60), 30 min–< 1 h (45/60), 1–< 2 h (1.5), 2–< 3 h (2.5), 3–< 4 h (3.5), and ≥ 4 h (4.0). Leisure-time physical activity was estimated by multiplying the frequency, average duration, and assigned MET value together for each activity. The

total physical activity was calculated by summing all daily and leisure-time physical activities. The questionnaire was based on a similar one with considerable validity and reliability, which was used in a Japan Public Health Center-based prospective study [1].

Variable preprocessing for data envelopment analysis

We transformed each variable using bestNormalize function in bestNormalize package employing a leave-one-out cross-validation procedure [2]; nutritional factors (n3PUFA, n6PUFA, SDF, and IDF) by ordered quantile normalization transformation; alcohol intake by standardized double reversed logarithmic transformation [ε = (maximum alcohol intake - minimum alcohol intake)/10, $\log_{10}$(maximum alcohol intake - minimum alcohol intake + 2ε) - $\log_{10}$(maximum alcohol intake - alcohol intake + 2ε)]; daily number of smoked cigarettes (smoking status) by standardized logarithmic transformation [$\log_{10}$(smoking status + 0.001)]; sleep time by standardized inverse hyperbolic sine (arcsinh) transformation [ln{sleep time + $\sqrt{}$(sleep time^2 + 1)}]; *Bacillus* by square-root transformation ($\sqrt{}$*Bacillus*); *Bifidobacterium*, *Streptococcus* and Faith's phylogenic diversity by ordered quantile normalization transformation; and *Lactobacillus* and *Lactococcus* by standardized logarithmic transformation [log10(*Lactobacillus* or *Lactococcus* + 0.001)]. After transformation, we added the sum of the lowest and second-lowest values within each variable to ensure non-negativity of DEA [3, 4]. These preprocessing steps ensured that all variables were appropriately scaled and normalized for use in DEA, allowing for the calculation of accurate efficiency across diverse input and output measures.

Sensitivity analysis of DEA

We calculated the efficiency scores of different models for assessing the robustness of our data. Input and output variables used in the sensitivity analysis are shown in

Supplementary Table 1.

References

1. Kikuchi H, Inoue S, Odagiri Y, Ihira H, Inoue M, Sawada N, et al. Intensity-specific validity and reliability of the Japan Public Health Center-based prospective study-physical activity questionnaire. Prev Med Rep. 2020;20:101169; doi: 10.1016/j.pmedr.2020.101169.
2. Ryan AP. Finding Optimal Normalizing Transformations via bestNormalize. The R Journal. 2021;13(1):310-29; doi: 10.32614/RJ-2021-041.
3. Boakye G, Li Y, Asare E. Determination of the Efficiency of Port Performance and Productivity Based on Data Envelopment Analysis in the West Africa Sub-region. Indian Journal of Science and Technology. 2021;14(46):3396-406; doi: 10.17485/IJST/v14i46.1755.
4. Kant P, Gupta S: Data Envelopment Analysis (DEA) Approach to Assess the Efficiency of Wholesale Markets in Various Cities of India. In: Proceedings of the Fifth International Conference of Transportation Research Group of India. Edited by Parida M, Maji A, Velmurugan S, Das A. Singapore: Springer Nature Singapore; 2022: 329-41.

Table S1. Variables used in each sensitivity analysis models.

Variables	Main model	Model 2	Model 3	Model 4	Model 5
**Inputs**					
n3PUFA	✓	✓	✓		
Soluble dietary fiber	✓	✓	✓	✓	
Insoluble dietary fiber	✓	✓	✓	✓	✓
Alcohol intake (unit/day)*	✓	✓	✓	✓	✓
Smoking	✓		✓		
Sleep time (h/day)	✓		✓		
**Output variables**					
Bacillus	✓	✓	✓	✓	✓
Lactobacillus	✓	✓	✓		
Lactococcus	✓	✓			
Streptococcus	✓	✓	✓	✓	✓
Faith's phylogenetic diversity	✓	✓	✓	✓	✓

n3PUFA, omega-3 poly-unsaturated fatty acids.

* One unit corresponds to 21 g alcohol.

Table S2. Results of sensitivity analysis of Tobit regression analysis on efficiency score

	Univariate analysis			Multivariate analysis		
	Coefficients	Standard error	P value	Coefficients	Standard error	P value
Age	0.86	0.42	0.043	-		
Sex (female)	-56.93	12.70	< 0.001	-36.10	13.50	0.008
BMI (kg/m^2)	3.65	1.63	0.026	3.52	1.69	0.038
eGFR (20 ml/min/1.73m^2)	-13.55	6.66	0.042	-		
Hb (mg/dl)	0.25	3.73	0.946	-10.84	4.33	0.012
BUN (mg/dl)	-0.07	0.23	0.757	-		
HbA1c (%)	1.59	9.70	0.869	-		
Total energy intake (500 kcal/day	22.74	7.64	0.003	0.04	0.01	0.012
Fat intake (g/day)	-1.49	0.52	0.004	-1.21	0.44	0.006
Salt intake (g/day)	-6.76	4.77	0.157	-8.42	4.10	0.040
Food frequency (times/day)						
Yogurt	32.28	12.41	0.009	-		
Natto*	19.16	14.02	0.172	-		
Sewead	-15.15	18.14	0.403	-		
Fried foods	4.29	29.49	0.884	-		
Coffee	-1.07	6.31	0.865	-		
Taking antibiotics (yes)	54.60	88.57	0.538	-		
Taking probiotics (yes)	-44.08	38.76	0.255	-		

Coefficients and standard errors are multiplied by 103, where 1.0 corresponds to 0.001 of the efficiency score. Variables with hyphens are dropped in the variable selection procedure.

BMI, body mass index; eGFR, estimated glomerular filtration rate; HbA1c, glycated hemoglobin A1c; BUN, blood urea nitrogen.
* Fermented soybeans.

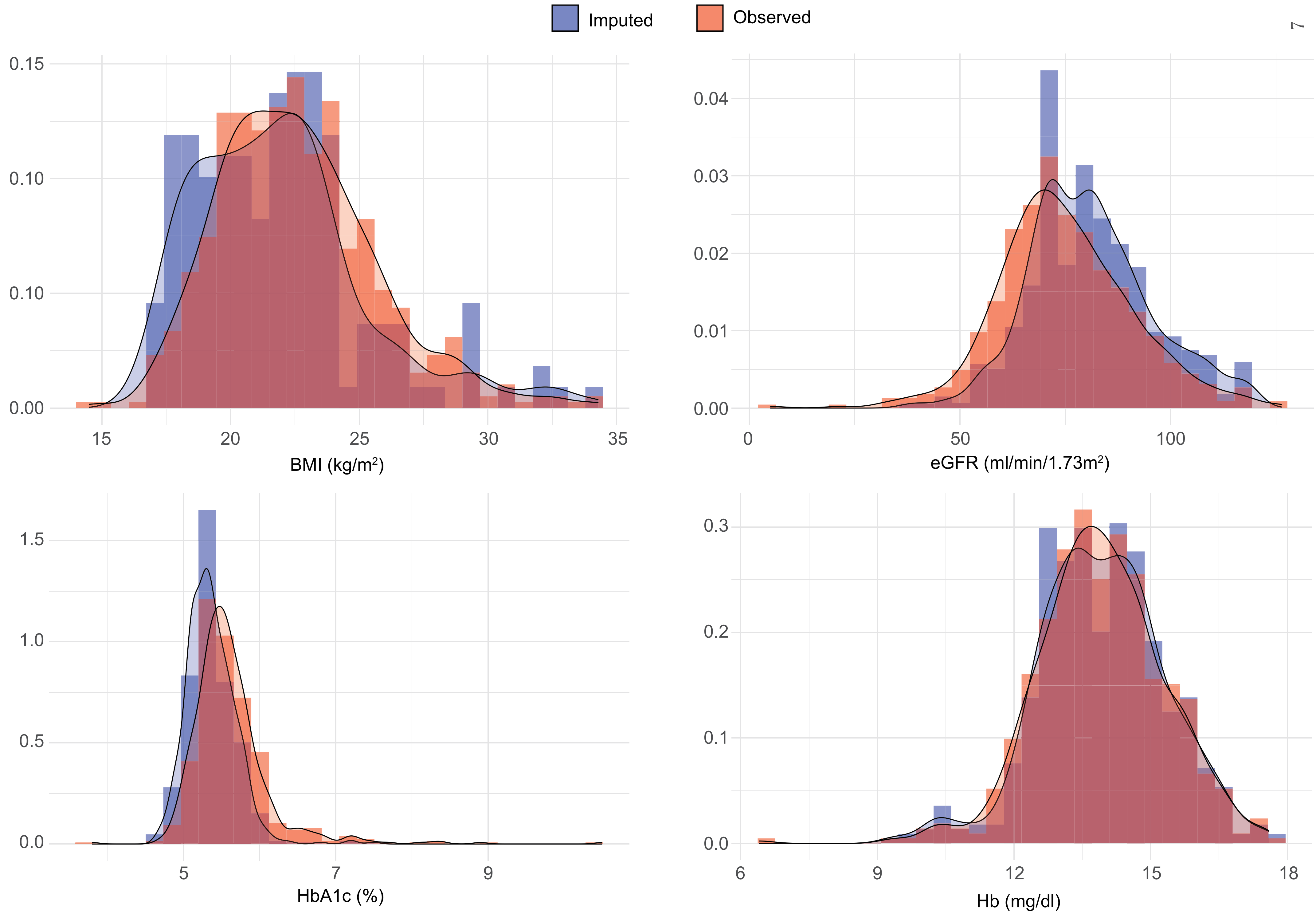

Supplementary Figure 1. Distribution of observed vs imputed values.

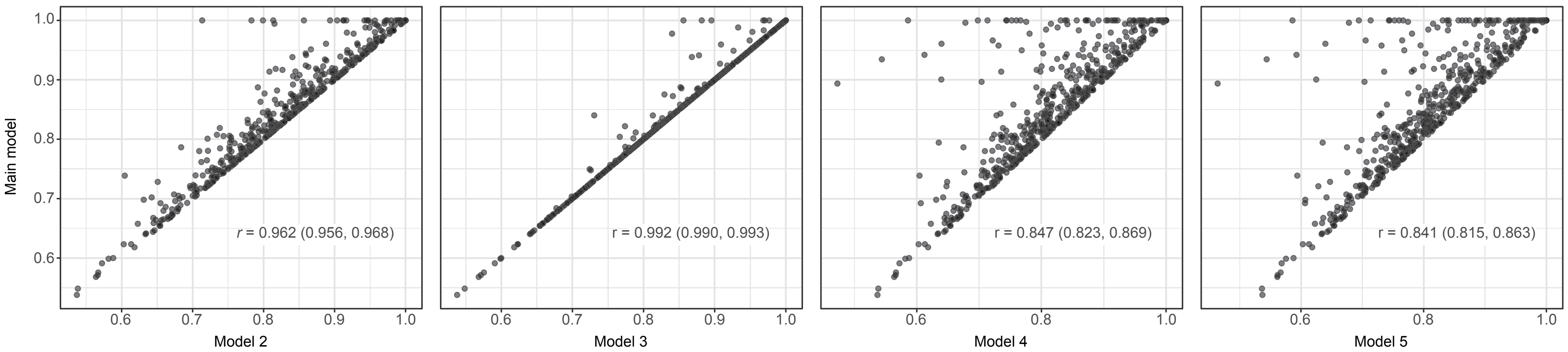

Supplementary Figure 2. Comparison of efficiency scores among five models of the DEA.

Correlations between the efficiency score of the main model and each alternative models were calculated using Pearson's product-moment corralation. 95% confidence intervals are shown in the parenthesis.
